# RELATCH: relative optimality in metabolic networks explains robust metabolic and regulatory responses to perturbations

**DOI:** 10.1186/gb-2012-13-9-r78

**Published:** 2012-09-26

**Authors:** Joonhoon Kim, Jennifer L Reed

**Affiliations:** 1 Department of Chemical and Biological Engineering, University of Wisconsin-Madison, Madison, WI, USA; 2 DOE Great Lakes Bioenergy Research Center, University of Wisconsin-Madison, Madison, WI, USA

## Abstract

Predicting cellular responses to perturbations is an important task in systems biology. We report a new approach, RELATCH, which uses flux and gene expression data from a reference state to predict metabolic responses in a genetically or environmentally perturbed state. Using the concept of relative optimality, which considers relative flux changes from a reference state, we hypothesize a relative metabolic flux pattern is maintained from one state to another, and that cells adapt to perturbations using metabolic and regulatory reprogramming to preserve this relative flux pattern. This constraint-based approach will have broad utility where predictions of metabolic responses are needed.

## Background

Computational modeling of metabolic networks has been useful in studying microbial metabolism and developing tools for many applications. Among different computational approaches, constraint-based models utilize genome-scale metabolic networks to predict metabolic flux distributions in microbial cells, and they have been used to guide metabolic engineering [1], drug discovery [2], and adaptive evolution [3] studies. For example, flux balance analysis (FBA) predicts metabolic flux distributions in optimally growing microbes, by maximizing biomass yields [4,5]. FBA can also predict the effects of gene deletions on metabolic behaviors by removing the associated reactions from the network, and its predictions are shown to be consistent with experimental observations for parental and gene knockout strains of *Escherichia coli *that have undergone adaptive evolution [6,7]. Recently, FBA has been used to discover drug targets by identifying essential metabolic functions in different growth conditions representing the host environment [8,9]. Incorporation of additional molecular crowding constraints, which restrict total enzyme levels and thus flux capacities, into FBA improves growth rate predictions of parental and mutant *E. coli *strains in different environmental conditions [10]. Genomics-driven constraints, such as grouping reaction constraints [11] have also been incorporated to improve flux and growth rate predictions in genetically or environmentally perturbed strains.

Several constraint-based approaches for integrating omics data (for example, transcriptomics, proteomics, or metabolomics) with metabolic models have been developed to predict metabolic flux distributions in different environmental or genetic conditions [12-16]. For example, E-Flux uses relative gene expression levels to place upper and lower bounds on individual fluxes in the models [12], while another approach, gene inactivity moderated by metabolism and expression (GIMME), instead uses expression data in the objective function to penalize the use of fluxes based on the magnitude of the flux and how far a gene's expression falls below a chosen threshold [14]. Another approach, integrative omics-metabolic analysis (IOMA), was developed to predict flux distributions by integrating proteomic and metabolomic data into kinetic constraints that are included in the models [13]. Unlike FBA, all of these omics-based approaches require data from the genetically or environmentally perturbed states to predict fluxes in that state, which is often not available ahead of time.

Since FBA assumes that cells grow optimally, other approaches are used to predict the behavior of perturbed strains that exhibit suboptimal growth (for example, unevolved mutants) due to regulatory restrictions or other metabolic limitations, without requiring any data from the perturbed state. One such approach is minimization of metabolic adjustment (MOMA), which predicts the behaviors of unevolved mutants by minimizing the sum of squared differences in flux distributions between mutant and parent strains [17]. MOMA has been used to improve production of valuable biochemicals [18,19], study epigenetic interactions associated with genetic diseases [20], and describe cooperative interactions between microbes [21]. Another approach, regulatory on/off minimization (ROOM) minimizes the number of significant flux changes in mutant strains relative to the parental strain [22]. Both MOMA and ROOM use a reference (for example, wild type) flux distribution to predict a perturbed flux distribution by minimizing the Euclidean and Hamming distances, respectively, and hence the reference flux distribution significantly affects their predictions. The reference flux distribution is normally determined in both approaches by FBA; however, a more accurate description of the reference state can be obtained from available experimental data as proposed here. Neither MOMA nor ROOM consider flux fold changes in their minimization procedures, and so large fold changes in flux may be predicted if the Euclidean or Hamming distances are minimized. While flux predictions with MOMA and ROOM show good correlation to experimental measurements [17,22] they can still be quantitatively inaccurate, which may guide valuable experimental efforts in the wrong direction. Therefore, there is still a need for a more accurate approach that accounts for metabolic and regulatory adjustments at a genome-scale to predict flux distributions in genetically and environmentally perturbed microbial systems, without requiring experimental data from perturbed states.

**Figure 1:**
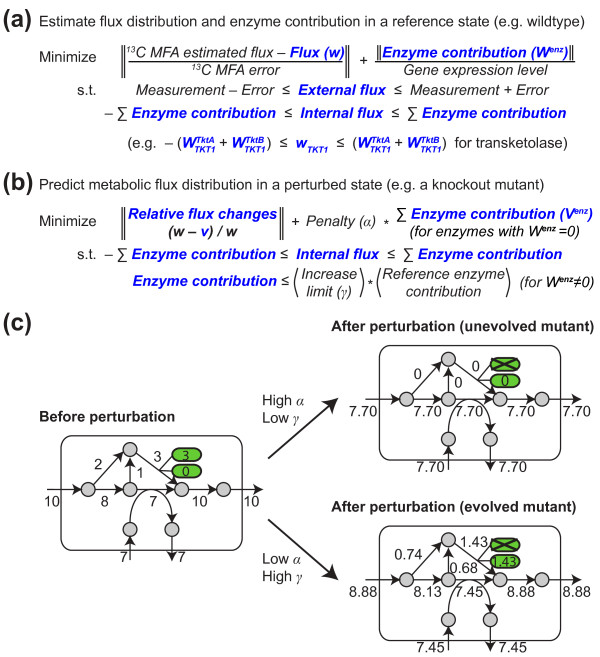
**Overview of RELATCH framework for predicting metabolic flux distributions in perturbed microbial systems**. **(a) **The metabolic flux distribution (*w*) and enzyme contributions (*W^enz^*) in a reference state are first estimated using metabolic flux analysis (MFA), physiological measurements, and gene expression data. Variables are shown in bold blue letters and experimental measurements are shown in plain letters. **(b) **The metabolic flux distribution (*v*) in a perturbed state is predicted by minimizing the relative flux changes and latent pathway activation from the reference state. Two parameters are used to represent the extent of adaptation to perturbations, a penalty for latent pathway activation (α) and a limit on contribution increase in active enzymes (γ). **(c) **An illustrative example of using RELATCH to predict the effects of deleting an isozyme, where contributions of two isozymes are shown in green.

In this work, we report a new approach, RELATCH for RELATive CHange (Figure 1), which estimates fluxes in a reference state and predicts perturbed flux distributions with greater quantitative accuracy than existing approaches both before and after adaptation to perturbations. Using the concept of relative optimality based on relative flux changes with respect to a reference flux distribution, we hypothesize that a relative metabolic flux pattern is preserved by the network structure for metabolic stability. The key assumptions of RELATCH are that perturbed strains would initially minimize relative metabolic changes within limited regulatory adjustments, and that they could further increase the capacity of previously active and inactive pathways as they adapt to perturbations. In this approach, we first utilize ^13^C metabolic flux analysis (MFA) results, physiological measurements, and gene expression data to approximate the genome-scale flux distribution and corresponding enzyme contribution in a reference state (for example, parental strain). Here, enzyme contribution refers to the flux a particular enzyme contributes towards the total flux through a reaction when contributions from all isozymes are considered. The gene expression data allow higher enzyme contributions for more highly expressed genes. Next, the flux distribution for a perturbed strain (for example, a knockout mutant) is predicted by minimizing relative flux changes and latent pathway activation (that is, when a previously inactive pathway becomes active) from the reference state, without requiring any data from the perturbed state. We have applied RELATCH to predict flux distributions in genetically or environmentally perturbed *Escherichia coli, Saccharomyces cerevisiae*, and *Bacillus subtilis *strains, and compared the model predictions to experimental data regarding fluxes, enzyme activity, gene expression, and genetic mutations. RELATCH predicts the metabolic behaviors of both unevolved and evolved knockout mutants and environmentally perturbed strains with significantly greater accuracy than existing approaches (with up to 100-fold decrease in the sum of squared errors between predicted and observed fluxes). Moreover, important metabolic and regulatory changes predicted by RELATCH are highly consistent with experimental observations, including gene expression, enzyme activity, and whole-genome resequencing. As a highly accurate tool for predicting metabolic flux responses to perturbations, we believe RELATCH can improve our understanding of changes that occur over adaptive evolution and how metabolism responds to perturbations.

## Results

To predict the flux distributions in perturbed systems, we employed two parameters - a penalty (α) for latent pathway activation and a limit on enzyme contribution increases (γ) for active enzymes (see Materials and methods). The underlying rationale for the RELATCH parameters is that strains would initially adjust to new perturbations with limited metabolic and regulatory adjustments, whereas over time they could adapt to these conditions by increasing the capacity of previously active and latent pathways. Here, we considered the recent loss of metabolic enzymes (for example, unevolved knockout mutants) or a change in growth conditions that cells are less accustomed to (for example, galactose) as conditions the cells are not adapted to. For these non-adapted conditions, we used tight parameter values, including a high penalty for latent pathway activation (α = 10) and restricted enzyme contribution increases in active enzymes from the parental state (γ = 1.1). We considered cells to be adapted to a condition if knockout strains were adaptively evolved, if parental strains were grown in conditions cells were accustomed to (for example, anaerobic growth), or if strains were grown in a chemostat (since strains grown in chemostat can rapidly accumulate beneficial mutations during the pre-culture and stabilization period [23]). For these adapted conditions, we used relaxed parameter values, including a low penalty for latent pathway activation (α = 1) and no restriction on enzyme contribution increases in active enzymes (effectively, γ = ∞). These two sets of parameter values were determined by analyzing four knockout *E. coli *strains before and after adaptive laboratory evolution described in the next section (Additional files 1 and 2) and were applied systematically to the other datasets.

### Flux predictions in *E. coli *mutant strains before and after adaptive laboratory evolution

We first used RELATCH to predict flux distributions in knockout mutants of *E. coli *K-12 MG1655 (*Δpgi, Δppc, Δpta*, and *Δtpi*) before and after adaptive laboratory evolution. MFA flux values for the parental strain were taken from an existing study [24] and mapped onto corresponding reactions in the iAF1260 metabolic model [25]. Gene expression data for the parental *E. coli *strain grown aerobically in glucose minimal medium from an earlier study [26] and MFA estimates were then combined to estimate the genome-scale flux distribution and enzyme contribution in the parental strain. This reference flux distribution was then used to predict flux distributions in unevolved and evolved knockout mutants and compared to the reported MFA flux estimates for these mutants by calculating the sum of squared errors per flux (SSE; Equation 6) and Pearson's correlation coefficient (r) [24].

**Figure 2:**
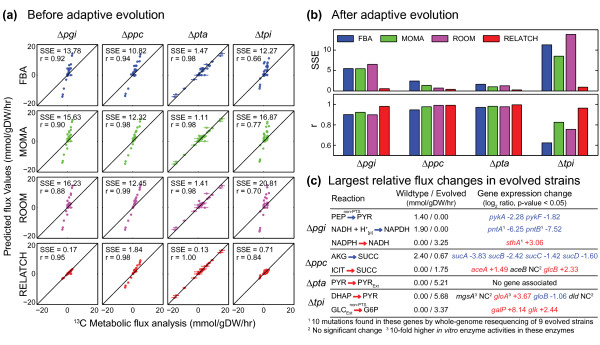
**Comparison of MFA estimated flux values and predicted flux values by different methods for four *E. coli *knockout mutants before and after adaptive evolution. (a) **The MFA estimated (x-axis) and predicted (y-axis) flux values, sum of squared errors per flux (SSE) and Pearson's correlation coefficient (r) are shown for each unevolved mutant. Error bars indicate the confidence intervals from MFA. The RELATCH predictions were made using the tight parameter values. **(b) **SSE and r values for different approaches used to predict behaviors for evolved mutants, where the average MFA results (across two independently evolved strains) were used to calculate the SSE and r values. The RELATCH predictions were made using the relaxed parameter values. **(c) **Largest relative flux changes predicted by RELATCH in evolved mutant strains. The relative flux changes, determined as ratios of predicted mutant fluxes to wild-type fluxes, were rank-ordered to identify reactions with the largest changes, which were then compared to experimental data, including enzyme activity, gene expression [24], and genetic mutations [27]. AKG, 2-oxoglutarate; DHAP, dihydroxyacetone phosphate; G6P, glucose-6-phosphate; GLC_EXT_, extracellular glucose; ICIT, isocitrate; PEP, phosphoenolpyruvate; PYR, pyruvate; SUCC, succinate.

RELATCH accurately predicted the metabolic flux distributions in four knockout mutants before undergoing adaptive evolution, while existing methods (FBA, MOMA, and ROOM) often over-predicted the flux values (Figure 2a). RELATCH effectively predicted the initial responses to the genetic perturbations, including the limited use of latent pathways and rerouting of fluxes around the metabolic defect, with an overall effect of decreasing network throughput. Only RELATCH predicted a significant reduction of growth and glucose uptake rates in three unevolved mutants (*Δpgi, Δppc*, and *Δtpi*), as well as slight increase in glucose uptake rate for the *Δpta *mutant (Additional file 3). In the *Δpgi *mutant, activation of the glyoxylate shunt, decreased flux through lower glycolysis (approximately 23% of the parental strain), and unaltered flux through pentose phosphate and Entner-Doudoroff pathways were successfully predicted by RELATCH. Activation of the glyoxylate shunt was also accurately predicted for the *Δppc *mutant, enabling an alternative mechanism for synthesizing citric acid cycle intermediates. The most notable initial response in the *Δpta *mutant was secretion of pyruvate instead of acetate, which was only predicted by RELATCH (MOMA and ROOM predicted acetate production similar to the parent strain using alternative acetate production routes). RELATCH's pyruvate secretion prediction in the *Δpta *mutant is due to the minimization of relative flux changes, since lowering the penalty for latent pathway activation and/or removing enzyme contribution constraints still led to pyruvate secretion predictions. In agreement with the MFA measurements and *in vitro *enzyme assays [24], RELATCH predicted activation of methylglyoxal pathway with limited capacity in the *Δtpi *mutant. Interestingly, RELATCH predicted no growth for the *Δppc *and *Δtpi *mutants when a very high penalty for latent pathway activation was used (Additional file 2), suggesting that the activation of latent pathways is needed to enable growth in these mutants.

For adaptively evolved knockout mutants, RELATCH also predicted mutant flux distributions (Figure 2b,c) and growth and glucose uptake rate recovery (Additional file 4) with the greatest quantitative accuracy. We should note that the predicted unevolved and evolved mutant flux distributions are different for RELATCH (due to use of tight and relaxed parameter values), while they are the same for the other three methods. The relaxed parameter values allowed further increases in enzyme contributions for active enzymes, as well as increased use of latent pathways, in order to compensate for the gene deletion. For the evolved *Δpgi *mutant, RELATCH predicted further increase in fluxes through the NADPH-producing pentose phosphate pathway and subsequent conversion of excess NADPH to NADH via NAD(P)H transhydrogenase (catalyzed by SthA), as compared to the unevolved mutant. This suggests that redox balancing is a bottleneck in the unevolved *Δpgi *mutant, which is consistent with a previous study [27] where whole-genome resequencing of evolved *Δpgi *strains found frequent mutations in the SthA (converting NADPH to NADH) and PntAB (converting NADH to NADPH) transhydrogenases. RELATCH also predicted a decrease in pyruvate kinase flux, which is consistent with gene expression data [24]. Interestingly, one of the two evolved *Δpgi *strains (E1) had slower growth and considerably higher acetate secretion compared to the other evolved *Δpgi *strain (E2) (Additional file 4), indicating that the *Δpgi *E1 strain is less optimal with respect to growth than *Δpgi *E2. It seems that the *Δpgi *E1 strain had evolved to a local optimum since another study showed that ten independently evolved *Δpgi *strains had similar growth and acetate secretion rates as the *Δpgi *E2 strain [27]. The flux values predicted by RELATCH were closer to the flux values in the *Δpgi *E2 strain (SSE = 0.32) than the flux values in the *Δpgi *E1 strain (SSE = 1.59). For the evolved *Δppc *and *Δpta *mutants, all methods predicted the flux patterns well (Figure 2b), but the extracellular fluxes and growth rates were most accurately predicted by RELATCH (Additional file 4). Increased glyoxylate shunt flux and decreased citric acid cycle flux from isocitrate to succinate in the evolved *Δppc *mutant were also correctly predicted by RELATCH, which is consistent with expression changes in the genes involved in these pathways [24]. For the *Δtpi *mutant, FBA incorrectly predicted high fluxes through the Entner-Doudoroff pathway to maximize biomass production, while RELATCH instead predicted increased use of the methylglyoxal pathway to produce pyruvate, and activation of the non-phosphotransferase system glucose transporters due to the decreased production of phosphoenolpyruvate. In this case, the RELATCH prediction agreed with MFA, enzyme activity, and gene expression measurements [24].

### Robustness of the flux predictions

We performed multiple sensitivity analyses using the same four *E. coli *mutant strains to demonstrate the robustness of RELATCH predictions. First, we investigated the effect of the reference flux distribution on predictions using four scenarios where less experimental data from the reference state is used. The results indicate that including more experimental data to estimate the reference flux distribution improves the accuracy of predictions by RELATCH, MOMA, and ROOM (Additional file 5). In all four scenarios, RELATCH outperformed existing approaches regardless of the availability of experimental data. Second, we investigated how the flux predictions change when different metabolic models of *E. coli *are used (iJR904 [28], iAF1260 [25], and iJO1366 [29]). The predictions were not sensitive to the metabolic models, except for ROOM predictions for the *Δpgi *mutant, and RELATCH predictions were still more accurate with all three models (Additional file 6). Third, we performed a sensitivity analysis of the two parameters (α and γ) in RELATCH, where we varied the values of the parameters and investigated their effects on the accuracy of flux predictions (Additional file 1 for details). The results indicate that the predictions were not significantly affected by the parameter values as long as α and γ were in the same order of magnitude (Additional file 2).

We also investigated the effects of alternative optimal solutions on the flux predictions. We first found alternative reference flux distributions by solving the reference flux estimation problem (Additional file 1, Equations S1 to S6) and subsequently minimizing and maximizing each flux variable with the fixed optimal objective function value. Among the 2,383 reactions in the iAF1260 model, only 80 total reactions had flux variability in the reference state, with 35 reactions (mostly in the nucleotide salvage pathway) having variability between 0.01 and 0.22 mmol/gDW/h and 45 reactions (mostly transporters) having variability less than 0.01 mmol/gDW/h. The variability is due to the redundancy and multi-functionality of enzymes in the nucleotide salvage pathway and multiple transport reactions, and is much lower than is often found across alternative FBA solutions (90 reactions with flux variability more than 1 mmol/gDW/h and 134 total reactions with variability). We subsequently used ten alternative reference state solutions from the five reactions with the most variability to predict the flux distributions in the unevolved and evolved four *E. coli *mutant strains, but we did not find any instances where the alternative reference states affected prediction errors (SSE values were the same). We also investigated the existence of alternative optimal solutions in perturbed states using the four *E. coli *mutant strains. We solved the flux prediction problem (Additional file 1, Equations S7 to S14) given a fixed reference state, and subsequently minimized and maximized each flux variable after fixing the optimal objective function value. The reactions included in the MFA dataset (whose experimental flux values are available for comparison) did not have any flux variability, which was also confirmed by the fact that slightly increasing or decreasing each flux value led to an increase in the objective function value. Together, these results indicate that there is little variation across alternative solutions for the reference state and that alternative solutions for the reference and perturbed states would likely result in similar SSE values.

### Growth rate and phenotype predictions for single knockout *E. coli *mutants

**Figure 3:**
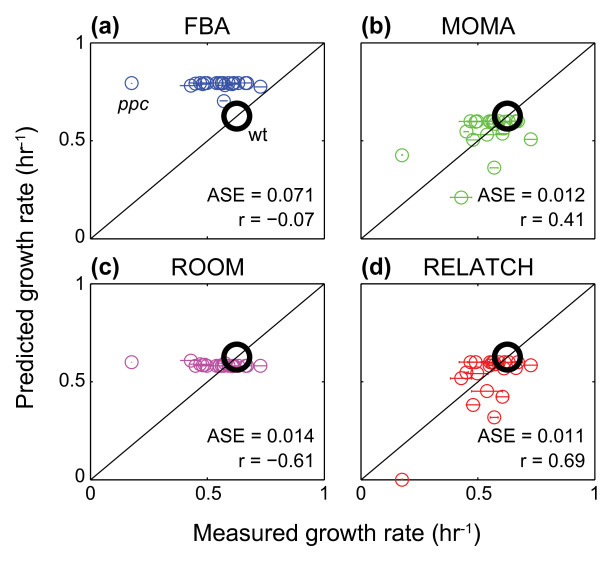
**Growth rate predictions for 22 single knockout *E. coli *mutants using FBA, MOMA, ROOM, and RELATCH with the tight parameter values. (a) **FBA, **(b) **MOMA, **(c) **ROOM, **(d) **RELATCH. The large black circles represent the growth rate of the parental strain. The average squared errors across all mutant (ASE; units are hr^-2^) and the Pearson's correlation coefficient (r) are shown for each method, and the horizontal error bars indicate the standard deviations across the triplicate experiments. Wt, wild type.

To further evaluate the accuracy of the different methods for predicting growth rates, we subsequently grew 22 single knockout mutants of *E. coli *K-12 BW25113 in glucose minimal medium (Additional file 1), and compared the measured growth rates to the predictions by RELATCH and other existing methods. To better estimate the flux distribution in the parental strain, we used MFA measurements and physiological parameters from a different study [30] where glucose uptake, acetate secretion and growth rates were more consistent with our parental strain growth curves. MOMA and RELATCH predictions were closest to experimentally observed growth rates (each with an average squared error across all mutants of 0.01 h^-2^), while FBA and ROOM generally predicted higher growth rates as noted in a previous study [22] (Figure 3; Additional file 7). The growth rate predictions for the *Δppc *mutant differed significantly among the four methods where FBA, MOMA, and ROOM predicted higher growth rates and RELATCH predicted no growth (note the RELATCH growth predictions differ from earlier *Δppc *predictions because the reference states differ). The growth of a *Δppc *mutant requires suppressor mutations in the glyoxylate shunt [31] and RELATCH predicted no growth due to inactivation of the glyoxylate shunt. If the glyoxylate shunt was activated in this mutant, then RELATCH would predict positive growth (0.41 h^-1^).

We also performed growth phenotype predictions for 1,260 *E. coli *single gene knockout strains. We compared the growth phenotype predictions by FBA, MOMA, and RELATCH to the list of experimentally essential genes from the iAF1260 study [25]. For RELATCH and MOMA, we used the reference flux distribution determined for Figure 2. The results show that RELATCH found more true negative cases than FBA without any additional false negative cases (Table 1). Although MOMA found six more true negative cases, it also had nine more false negative cases (Additional file 8 for details).

**Table 1:** A summary of growth phenotype predictions for 1,260 single gene knockout *E. coli *mutants

(Experimental/model)	FBA	MOMA	RELATCH
+/+	993	984	993
+/-	29	38	29
-/+	81	75	78
-/-	157	163	160
Accuracy (%)	91.27	91.03	91.51

The RELATCH predictions were made using the tight parameter values. A plus sign (+) indicates growth and a minus sign (-) indicates no growth.

### Predicting metabolic responses to the complete and partial loss of metabolic functions

Next, we used RELATCH to predict the effects of gene knockouts, resulting in the total or partial loss of reaction activities, in three different organisms. Existing methods like FBA, MOMA, and ROOM cannot predict the effects of isozyme deletions since they are based on reaction deletions; however, RELATCH can estimate the flux each isozyme contributes towards the total flux through a reaction and predict the consequences of removing individual isozymes. Large-scale MFA datasets for parental and knockout strains of *E. coli, S. cerevisiae*, and *B. subtilis *were taken from previous studies [23,32,33], and gene expression datasets for parental strains grown aerobically in glucose minimal medium were obtained from the same study if available [23] or related studies [34,35].

**Figure 4:**
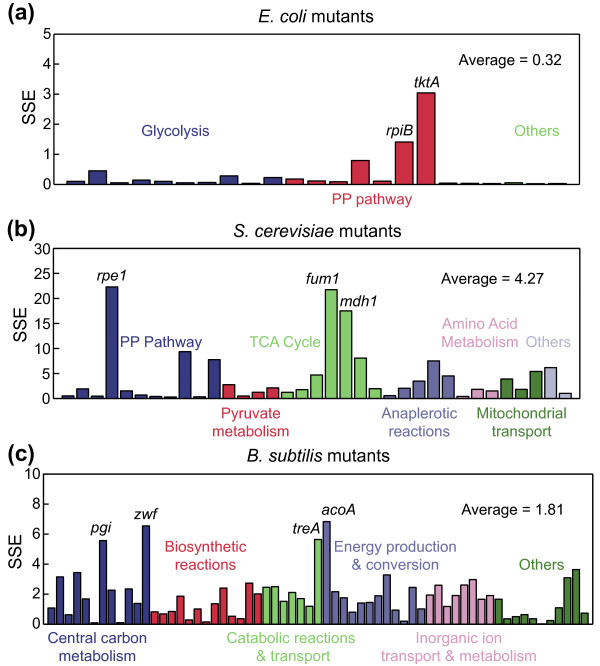
**Prediction of metabolic flux distributions in knockout mutants of *E. coli, S. cerevisiae*, and *B. subtilis*. (a) ***E. coli*, **(b) ***S. cerevisiae*, **(c) ***B. subtilis*. The sum of squared errors per flux (SSE) is shown for each mutant strain, and the average SSE across all mutants is shown for each organism. The RELATCH predictions were made using the relaxed parameter values for (a) and the tight parameter values for (b,c) since the strains were grown in a chemostat and batch cultures, respectively. PP, pentose phosphate, TCA, tricarboxylic acid cycle.

We first tested the capability of RELATCH to predict fluxes in *E. coli *mutants grown in chemostat cultures [23]. The results indicated that RELATCH can effectively predict the metabolic responses to the deletion of single enzymes, including isozymes, by cells grown in chemostat cultures (Figure 4a). For 21 out of 23 mutants, the SSE was less than 1 and the average SSE across all mutants was 0.32 (see Additional file 9 for details). Interestingly, the *ΔtktA *and *ΔrpiB *mutants had decreased biomass yields (approximately 65% and 70% of the parental strain's, respectively), which was not predicted by RELATCH. In *E. coli*, transketolase and ribose-5-phosphate isomerase are essential for growth on glucose [36,37], and *E. coli *has two isozymes for each (TktA/TktB and RpiA/RpiB, respectively). Since TktA is known to be responsible for the major transketolase activity [38], it seems that the activity of TktB alone in the *ΔtktA *mutant is not sufficient for efficient growth (the *ΔtktB *mutant had a similar behavior to the wild type's). However, the behavior of the *ΔrpiB *mutant was unexpected since RpiB is thought to be the minor isozyme under this growth condition [37], and RELATCH predicts a flux distribution similar to the parent strain for this mutant.

To examine whether these results were unique to *E. coli*, we subsequently used RELATCH to predict metabolic flux distributions in 35 knockout *S. cerevisiae *mutants of central metabolic genes grown in batch cultures [32] using the iMM904 metabolic model [39]. The predictions were in good agreement with experimental measurements for most mutants, including the mutants (for example, *zwf1Δ, ald6Δ, pda1Δ*, and *oac1Δ*) whose fluxes deviated significantly from the parental strain (Figure 4b; Additional file 9). Further investigation into a few mutants with less accurate predictions (*rpe1Δ, fum1Δ*, and *mdh1Δ*) was done to identify potential model improvements. The *rpe1Δ *mutant had the lowest measured glucose uptake rate among all 35 mutants (approximately 26% of parental value), but RELATCH predicted a larger glucose uptake rate (approximately 65% of parental value) and reversal of the second transketolase reaction (TKT2, so that erythrose 4-phosphate is produced). However, reversal of TKT2 was not experimentally observed at a statistically significant flux level in any of the 35 mutants. When the TKT2 reaction was constrained to be irreversible in the forward direction consuming erythrose 4-phosphate, the RELATCH prediction improved significantly for the *rpe1Δ *mutant (SSE decreased from 22.3 to 0.9), suggesting that the TKT2 reaction may proceed only in the forward direction in this condition, which is supported by a recent thermodynamic analysis study [40]. Two mutants in the TCA cycle, *fum1Δ *and *mdh1Δ*, experimentally had very similar flux distributions and impaired growth, but RELATCH predicted higher growth rates for these two mutants by using alternative routes involving the glyoxylate shunt and malic enzyme. Mdh1 is a component of malate-oxaloacetate and malate-aspartate shuttles, which regulate the NADH/NAD ratio in mitochondria and cytosol [41], and this regulation is not accounted for in the current metabolic model. Therefore, the transport of oxaloacetate could be affected in the *mdh1Δ *mutant, as well as the *fum1Δ *mutant, which would result in limited availability of mitochondrial malate. Interestingly, the *oac1Δ *mutant, which lacks the mitochondrial oxaloacetate transporter, also had a very similar flux distribution to the *mdh1Δ *and *fum1Δ *mutants experimentally, which supports the hypotheses that deletion of *mdh1Δ *and *fum1Δ *alters oxaloacetate transport activity. Overall, RELATCH predicted the metabolic responses to a number of genetic perturbations in yeast, as well as led to potential model improvements based on discrepancies (see Additional file 10 for a comparison of prediction methods for mutants that do not involve isozymes).

We also analyzed a large-scale MFA dataset for parental and mutant strains of *B. subtilis *grown in batch cultures [33]. In comparison to the MFA datasets for other organisms, the *B. subtilis *MFA results contained fewer flux estimates (four internal fluxes and two external fluxes), which could affect our metabolic flux distribution and enzyme contribution estimates for the reference state. Among the 137 viable mutants in the dataset, 63 of the deleted genes were in the iYO844 metabolic model [42] and the effects of these 63 deletions were predicted by RELATCH (see Additional file 10 for a comparison of prediction methods for mutants that do not involve isozymes). The predictions were consistent with the MFA measurements for most *B. subtilis *mutants involving different metabolic pathways, except for the *Δpgi, Δzwf, ΔtreA*, and *ΔacoA *mutants (Figure 4c; Additional file 9). The *B. subtilis Δpgi *mutant had significant flux through the pentose phosphate pathway, allowing the mutant to grow slightly slower than the parent strain (approximately 82% of growth rate), which is very similar to the behavior of the evolved *E. coli Δpgi *mutant [24]. The *Δzwf *mutant also exhibited a flexible response by increasing glycolytic flux and acetate production. The flux distributions for both of these two mutants were more accurately predicted if the relaxed RELATCH parameter values were used instead of the tight parameter values to account for the mutant's robust responses (SSE decreased from 5.57 to 1.28 for the *Δpgi *mutant and from 6.54 to 1.81 for the *Δzwf *mutant). The *ΔtreA *and *ΔacoA *mutants lack enzymes involved in trehalose and acetoin catabolism, respectively, which were both absent from the medium. RELATCH predicts these genes would be dispensable under the condition tested and it is unclear why these mutants exhibit a significant growth defect (reduced glucose uptake rate and growth rate).

### Predicting flux redistribution in response to environmental perturbations

**Figure 5:**
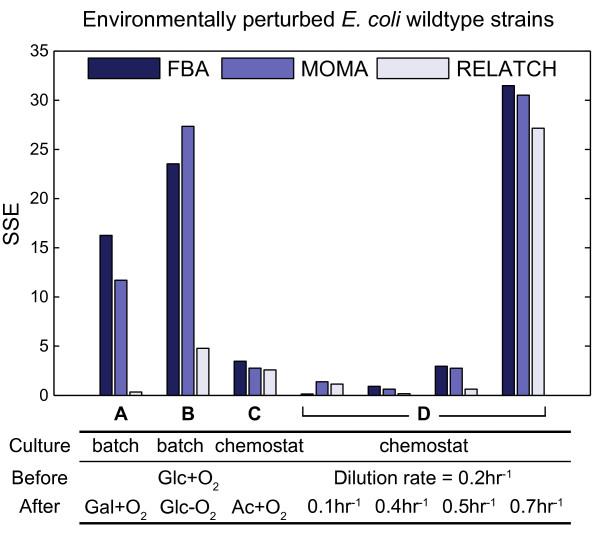
**Metabolic flux predictions by different methods in environmentally perturbed *E. coli *wild-type strains. (a-d) **The flux distribution of wild type grown aerobically on glucose in a batch (a,b) or chemostat (c,d) culture was used as a reference state to predict fluxes in galactose medium (a), glucose anaerobic condition (b), acetate medium (c), and chemostat (d) at different dilution rates. The RELATCH predictions were made using the tight parameter values for (a) or the relaxed parameter values for (b-d).

We further tested the ability of RELATCH, FBA, and MOMA to predict shifts in metabolic fluxes due to environmental perturbations, where cells are grown in different media or reactor conditions. First, we analyzed the metabolic flux distributions when cells move from glucose to galactose minimal medium in aerobic batch cultures [30]. In contrast to glucose, *E. coli *is not well adapted to and grows slowly on galactose, but can improve growth after undergoing adaptive laboratory evolution [43]. We used the MFA and expression data for the *E. coli *strain grown aerobically in glucose minimal medium [30] to predict flux distributions on galactose. The RELATCH prediction was much more accurate than the predictions from other methods, and the results suggest that galactose metabolism is limited by the capacity of galactose utilization pathways (Figure 5a). Interestingly, when the relaxed parameters were used instead of the tight parameters, the predicted flux distribution was close to experimental values for a NagC transcription factor mutant that had derepressed galactose uptake and thus increased growth rate [30] (Additional file 11).

Next, we predicted flux distributions when cells are grown anaerobically on glucose in batch culture [44]. *E. coli *is well adapted to anaerobic glucose catabolism since it is naturally found in the intestinal tract of mammals, but the metabolic flux distributions are quite distinct from those in aerobic conditions due to transcriptional regulation, and redox and energy metabolism differences. For this case, we used the MFA [44] and expression [26] data for the *E. coli *strain grown aerobically in glucose minimal medium for the reference state. In the MFA flux measurements, aerobic and anaerobic conditions were simulated using two different metabolic networks. Instead of modifying the metabolic network, here we compared the sum of the predicted pyruvate dehydrogenase (producing CO_2_) and pyruvate formate lyase (producing formate) fluxes to the measured flux from pyruvate to acetyl-CoA and production of C1 compounds. Surprisingly, RELATCH was able to predict an almost two-fold increase in glycolytic fluxes, as well as significant ethanol production in anaerobic conditions, in agreement with experimental data (Figure 5b). However, the favored use of pyruvate formate lyase over pyruvate dehydrogenase was not predicted by MOMA or RELATCH, indicating that additional transcriptional regulatory knowledge is needed to predict such behaviors.

We also tested RELATCH's capability to predict responses to carbon source changes (glucose to acetate) in chemostat cultures. Aerobically, *E. coli *grows efficiently on acetate as a sole carbon source (low growth rate but high biomass yield) and rapidly adapts from growth on glucose to acetate [45]. The metabolic flux distribution [46] and expression data [23] for *E. coli *BW25113 grown on glucose in chemostat with a dilution rate of 0.2 h^-1 ^were used to first estimate a glucose reference state. Since there are two acetate utilization pathways in *E. coli *(via Ack-Pta and Acs) and MFA cannot distinguish between them, we used the sum of the predicted fluxes through both pathways in our comparison (Figure 5c). The predictions by different methods were all similar to experimental observations; however, RELATCH accurately predicted use of the oxidative pentose phosphate pathway to make pentose phosphates, which is supported by a *zwf *gene knockout study [46], while FBA and MOMA predicted use of the non-oxidative pentose phosphate pathway. In addition to the glyoxylate shunt, RELATCH predicted use of the glycerate pathway (Gcl-GlxR-GlxK) feeding glyoxylate into glycolysis/gluconeogenesis, which is experimentally up-regulated during growth on acetate [47].

Finally, we analyzed the metabolic flux distributions when cells are grown in chemostat at different dilution rates (D). The MFA and expression measurements for *E. coli *BW25113 at D = 0.2 h^-1 ^[23] were used to estimate the reference state, and the MFA data at D = 0.1, 0.4, 0.5, and 0.7 h^-1 ^[23] were compared to predicted flux values. When the change was moderate (D = 0.1, 0.4, and 0.5 h^-1^), the model predictions were in good agreement with the data (Figure 5d). However, the experimental flux distribution at D = 0.7 h^-1 ^was significantly different from the others, including increased citric acid cycle flux and acetate production, which all three methods failed to predict. Also, the biomass yield in this condition was much lower, indicating cells were growing suboptimally due to acetate overflow.

## Discussion

Genome-scale metabolic models are being rapidly developed for many organisms, and their applications in biological discovery, metabolic engineering, evolution, and drug discovery continue to expand [48]. Constraint-based models and methods are useful tools to investigate the metabolic potential of an organism and predict its cellular behavior. These models describe the possible metabolic behaviors within given physicochemical constraints, but do not necessarily provide a single metabolic state of the system of interest. Based on an optimal growth assumption, FBA predictions are shown to be well correlated to experimental data for evolved cells [49], but are less accurate for the behaviors of unevolved cells, which can grow suboptimally. Alternatively, MOMA and ROOM were developed to predict such behaviors of unevolved genetically perturbed systems without requiring any data from a perturbed state. While these latter two methods show good correlation between predicted and observed fluxes, they are still limited in their ability to predict flux distributions with high quantitative fidelity. Also, intracellular flux distributions predicted by existing methods have not yet been rigorously evaluated against genetic perturbations in organisms besides *E. coli *or against environmental perturbations.

In this work, we presented a new approach, RELATCH, to predict the quantitative metabolic behaviors of genetically or environmentally perturbed microbial systems. RELATCH utilizes available information to estimate the metabolic state before perturbations (MFA, physiological, and transcriptomic measurements) and predict the effects of perturbations using two parameters whose values are chosen according to the nature of the perturbations. We demonstrated RELATCH's prediction capability using large-scale datasets from different perturbation experiments for three model organisms, including unevolved/evolved mutants, batch/chemostat cultures, and genetic/environmental perturbations. Our results show that RELATCH dramatically outperforms existing methods with regard to predicting intracellular flux distributions in gene knockout strains. In addition, RELATCH predictions for environmentally perturbed *E. coli *strains were significantly more accurate compared to other methods, especially for strains grown in batch cultures (approximately 5- to 50-fold lower SSE) where substrate uptake and growth rates are difficult to predict. It was previously suggested that *B. subtilis *maintains suboptimal metabolism for the sake of robustness, which led to flexible responses by maintaining the relative metabolic flux distributions [33]. This is consistent with our assumption that perturbed strains would have minimal relative changes in metabolic fluxes with limited regulatory adjustment. While not done here, it is possible that RELATCH predictions could be further improved if organism-specific parameter values were found by training the algorithm on a small dataset. Parameter differences, if identified, would characterize how organisms achieve metabolic robustness.

While the constraint-based metabolic models may not provide a detailed description of the dynamic metabolic and regulatory mechanisms in response to perturbations, they can still provide accurate snapshots of metabolic states during adaptation at a genome-scale level without the need for detailed kinetic parameters. The underlying metabolic and regulatory responses can be inferred from further analyses of the changes in metabolic flux distributions. We showed here that RELATCH can accurately describe such changes using two simple parameters - a penalty for latent pathway activation and a limit on enzyme contribution increases in active pathways. In addition to consistency with MFA datasets, RELATCH predictions were also consistent with previous experimental data, including transcriptomics (for example, increased expression of glyoxylate shunt in evolved *Δppc *mutants), enzyme assay (for example, increased activity of methylglyoxal pathway in *Δtpi *mutants), and whole-genome resequencing (for example, mutations in SthA and PntAB in evolved *Δpgi *mutants). Using RELATCH, the metabolic changes arising from adaptation to perturbations were postulated by probing the flux space using these two parameters. This allows for the generation of hypotheses regarding the importance of metabolic changes, which is useful for metabolic engineering or drug targeting.

## Conclusions

Given the ability of RELATCH to predict metabolic responses arising from perturbations with significantly greater quantitative accuracy, the approach can be used to improve the production of biofuels, therapeutics, and commodity chemicals, as well as to identify drug targets for human pathogens. Further integration of RELATCH into computational strain design approaches as a quadratic cellular objective is also possible [50]. In addition, RELATCH could potentially be used to predict metabolic flux distributions in higher eukaryotes (for example, plant or mammalian cells) where biological objective functions are not always obvious. With RELATCH's predictive accuracy, general applicability, and low data requirements, this computational approach will benefit a wide variety of fields.

## Materials and methods

### Estimating the flux distribution and enzyme contribution in a reference state

We introduced new variables for enzyme contributions ($W_{j,n}^{enz}$) for all reaction (*j*)-enzyme (*n*) pairs using gene-to-protein-to-reaction (GPR) associations. To estimate the flux distribution (*w*) and enzyme contributions (*W^enz^*) in a reference state, we utilized MFA measurements and gene expression data. The sum of squared differences between flux variables and MFA estimates (*w^exp^*) weighted by the reciprocal of confidence intervals (*w^conf^*), and the sum of squared enzyme contributions weighted by the reciprocal of enzyme expression values (*E_n_*) were minimized to calculate the flux distribution and corresponding enzyme contribution (Equation 1). Using Equation 1, isozymes with higher expression will have higher enzyme contributions unless the associated flux is zero. If a reaction *j *has known GPR ($j\in J_{GPR}$) and is associated with multiple isozymes, *N(j)*, the flux value through the reaction is constrained by the sum of enzyme contributions for all the associated isozymes (Equation 2). If multiple reactions are associated with a multi-functional enzyme, different *W^enz ^*variables are assigned to each enzyme-reaction pair. External flux values (for example, glucose uptake rates) were constrained using the physiological measurements from the reference state (see Additional file 1 for details):

(1) $$\underset{w,W^{enz}}{\text{min}}\;\underset{j\in J_{MFA}}{\sum }{\left(\frac{w_{j}^{exp}-w_{j}}{w_{j}^{conf}}\right)}^{2}+\underset{j\in J_{GPR}}{\sum }\underset{n\in N\left(j\right)}{\sum }\frac{{\left(W_{j,n}^{enz}\right)}^{2}}{E_{n}}$$

(2) $$-\underset{n\in N\left(j\right)}{\sum }W_{j,n}^{enz}\leq w_{j}\leq \underset{n\in N\left(j\right)}{\sum }W_{j,n}^{enz}\;\forall j\in J_{GPR}$$

### Predicting the flux distribution in a genetically or environmentally perturbed state

Using the estimated metabolic flux distribution (*w*) and enzyme contribution (*W^enz^*) in a reference state, the metabolic flux distribution (*v*) and enzyme contribution (*V^enz^*) in a perturbed microbial system was predicted by minimizing the relative flux changes from a reference state for reactions active in the reference state (*J_act_*) and the enzyme contribution increases for enzymes inactive in the reference state (*N\N_act_*) with a penalty α (Equation 3). In addition to enzyme contribution constraints on fluxes (Equation 4), we imposed a limit (γ) on contribution increases for active enzymes (*N_act_*) (Equation 5). We used two different sets of values for these parameters (tight parameter values of α = 10 and γ = 1.1, or relaxed parameter values of α = 1 and γ = ∞) when predicting metabolic behaviors to different perturbations (see Additional file 1 for details). To simulate cases where γ = ∞, Equation 5 is omitted since the *V^enz ^*values are not constrained by the *W^enz ^*values. A gene knockout was simulated by setting the contribution of associated enzymes to zero, and an environmental perturbation was simulated by changing what metabolites are allowed to be taken up.

(3) $$\underset{v,V^{enz}}{\text{min}}\;\underset{j\in J_{act}}{\sum }{\left(\frac{w_{j}-v_{j}}{w_{j}}\right)}^{2}+\alpha \underset{j\in J_{GPR}}{\sum }\underset{n\notin N_{act}\left(j\right)}{\sum }V_{j,n}^{enz}$$

(4) $$-\underset{n\in N\left(j\right)}{\sum }V_{j,n}^{enz}\leq v_{j}\leq \underset{n\in N\left(j\right)}{\sum }V_{j,n}^{enz}\;\forall j\in J_{GPR}$$

(5) $$V_{j,n}^{enz}\leq \gamma W_{j,n}^{enz}\;\forall j\in J_{GPR},\forall n\in N_{act}\left(j\right)$$

The estimated flux distribution (*w*) used by RELATCH was also used for MOMA and ROOM calculations. For RELATCH, MOMA, and ROOM calculations, none of the exchange fluxes (except for growth rates in the chemostat cases since these are known *a priori*) were constrained to the measured values in the perturbed states, but they were instead predicted by the methods. To evaluate the differences between model predictions (*v*) and experimental MFA results (*v^exp^*) we calculated the sum of squared errors per flux (SSE) using the following equation:

(6) $$SSE=\frac{\underset{j\in J_{MFA}}{\sum }{\left(v_{j}^{exp}-v_{j}\right)}^{2}}{\left|J_{MFA}\right|}$$

In all instances, only the *w *and *W^enz ^*from the reference state are used to estimate *v*, and none of the *v*^*exp *^fluxes were used to predict *v*. An implementation of RELATCH using the COBRA Toolbox for MATLAB [51] can be found in Additional file 12.

## Abbreviations

FBA: flux balance analysis; GPR: gene-to-protein-to-reaction; MFA: metabolic flux analysis; MOMA: minimization of metabolic adjustment; ROOM: regulatory on/off minimization; SSE: sum of squared errors per flux.

## Competing interests

The authors declare that they have no competing interests.

## Authors' contributions

JK participated in the design of the study, performed the computational analysis, and drafted the manuscript. JLR conceived of the study, participated in its design and coordination, and helped to draft the manuscript. All authors read and approved the final manuscript.

## Supplementary Material

Additional File 1 **Supplementary Materials and methods**. Click here for file

Additional File 2 **Supplementary Figure S1**. Sensitivity analysis of parameters using *E. coli *knockout strains (*Δpgi, Δppc*, and *Δtpi*) before and after adaptive evolution. Click here for file

Additional File 3 **Supplementary Table S1**. Comparison of predicted and experimentally measured values of growth, substrate uptake, and product secretion rates for four *E. coli *mutants before adaptive evolution. Click here for file

Additional File 4 **Supplementary Table S2**. Comparison of predicted and experimentally measured values of growth, substrate uptake, and product secretion rates for four *E. coli *mutants after adaptive evolution. Click here for file

Additional File 5 **Supplementary Figure S2**. Sensitivity analysis of data used to generate reference flux distributions. Click here for file

Additional File 6 **Supplementary Figure S3**. Sensitivity analysis of metabolic network models. Click here for file

Additional File 7 **Supplementary Table S3**. Growth rate predictions for 22 single knockout *E. coli *mutants. Click here for file

Additional File 8 **Supplementary Table S4**. Growth phenotype predictions for 1,260 single knockout *E. coli *mutants. Click here for file

Additional File 9 **Supplementary Table S5**. RELATCH prediction errors (SSE) for *E. coli, S. cerevisiae*, and *B. subtilis *knockout mutants shown in Figure 4. Click here for file

Additional File 10 **Supplementary Figure S4**. Comparison of metabolic flux predictions using RELATCH and MOMA for knockout mutants of *S. cerevisiae *and *B. subtilis*. Click here for file

Additional File 11 **Supplementary Figure S5**. Comparison of MFA estimated fluxes and predictions by RELATCH for *E. coli *strains grown on galactose. Click here for file

Additional File 12 **Implementation of RELATCH**. RELATCH is implemented using the COBRA Toolbox for MATLAB. Click here for file
