# Supplementary material for: RELATCH: relative optimality in metabolic networks explains robust metabolic and regulatory responses to perturbations
Source: Genome Biol. 2012 Sep 26;13(9):R78. doi: 10.1186/gb-2012-13-9-r78 (PMC3506949; doi:10.1186/gb-2012-13-9-r78)
Supplement: Additional File 1 — Supplementary Materials and methods. [file gb-2012-13-9-r78-S1.PDF]

Supplementary Materials and methods

Metabolic network models and constraint-based methods

The genome scale metabolic models of *E. coli* (iAF1260 [1]), *S. cerevisiae* (iMM904 [2]), and *B. subtilis* (iYO844 [3]) were used in this study. Since FBA solutions are not unique, parsimonious flux balance analysis (pFBA) was used to obtain FBA solutions which maximize biomass yields and minimize flux usage [4]. For FBA predictions of mutant strains grown in batch cultures, the glucose uptake rate was constrained to the parental strain's value, and growth rate was maximized with flux usage minimized. For FBA predictions of mutant strains grown in chemostat cultures, the glucose uptake rate and flux usage were minimized while growth rate was constrained to the dilution rate. For FBA predictions of environmental perturbations in batch cultures, the glucose uptake rate in the reference state was used for the substrate uptake rates, and growth rate was maximized with flux usage minimized (Fig. 5A and 5B). For FBA predictions of environmentally perturbed chemostat cultures, the substrate uptake rate and flux usage were minimized while growth rate was fixed to the dilution rate (Fig. 5C and 5D).

For MOMA, ROOM, and RELATCH predictions, the same parental strain's flux distribution estimated from ^{13}C MFA, physiological measurements, and gene expression data was used as a reference state instead of a FBA solution. The original quadratic objective function instead of a linear variant was used for MOMA, and the parameter values for flux prediction ($\delta = 0.03$ and $\varepsilon = 0.001$) from the original study [5] were used for ROOM. The exchange fluxes, including substrate uptake rates, were allowed to vary from the reference state for MOMA, ROOM, and RELATCH. All optimization problems were solved using CPLEX (for FBA and ROOM) or

CONOPT (for MOMA and RELATCH) accessed via GAMS 23.7 (GAMS Development Corp., Washington, DC, USA).

For all prediction methods, exchange reactions were excluded from the objective functions (i.e. changes in these reactions were not minimized). For *E. coli* analyses, spontaneous reactions (associated with s0001 pseudo-gene in iAF1260 model) and outer membrane transport reactions via diffusion ('Transport, Outer Membrane Porin' subsystem) were excluded from the objective functions as well. In addition, reactions involved in oxidative stress response (CAT, SPODM, and SPODMpp) or formate-hydrogen lyase (FHL) were constrained to zero, and the P/O ratio was constrained as described in the original iAF1260 study [1]. For the *Δpta* mutants [6], the EutD isozyme was assumed to be non-functional for phosphate acetyltransferase reaction. The *eut* operon is normally not expressed unless induced by ethanolamine and vitamin B12 [7]. If EutD is included then all existing methods (FBA, MOMA, and ROOM) predicted the same flux distribution as the parental strain since no reaction was removed by the *pta* deletion. For the *Δtpi* mutants [6], among multiple methylglyoxal pathways from dihydroxyacetone phosphate to pyruvate in the iAF1260 model, we used the methylglyoxal synthase and glyoxalase I pathway (MgsA-GloAB-Ldh) for comparison, which is the only fully annotated pathway, based on its high levels of enzyme activity and gene expression [6]. While the majority of reactions were associated with genes for the iAF1260 *E. coli* model (94%) and iYO844 *B. subtilis* model (89%), ~26% of the reactions in the iMM904 *S. cerevisiae* model were not associated with any gene. While genes or enzymes responsible for these reactions have not yet been identified, an enzyme should exist for a metabolic reaction to happen unless the reaction is spontaneous. Therefore, we assigned an artificial enzyme for each reaction without associated genes (except for spontaneous or exchange reactions) to account for the changes in the contribution of unknown enzymes. The

average of expression values was used for these unknown enzymes in the objective function when estimating the enzyme contributions in a reference state (Eq. 1). The exchange and spontaneous reactions were also excluded from the objective functions for *S. cerevisiae* and *B. subtilis* analyses.

Estimating the metabolic flux distribution and enzyme contribution in a reference state

The flux distribution for a parental strain was first estimated to provide a reference flux distribution (w) and enzyme contribution (W^{enz}) using MFA, physiological measurements, and gene expression data. The MFA estimated flux values (w^{exp}) and confidence intervals (w^{conf}) for reactions included in the MFA datasets (J_{MFA}) were used to define the first term of the objective function (Eq. S1), where the squared sum of differences between the MFA estimates and flux variables weighted by the reciprocal of the interval was minimized. The flux variables for reactions with gene to protein to reaction associations (J_{GPR}) were constrained by the sum of non-negative enzyme contribution variables for associated enzymes ($N(j)$) (Eqs. S2 and S3), and the sum of squared enzyme contributions weighted by the reciprocal of expression values (E_n) was minimized in the objective function in addition to the first term (Eq. S1). Measured flux values (w^{meas}) and standard deviations (w^{std}) for substrate uptake and product secretion rates were used to constrain the external fluxes (J_{ext}) (Eq. S4). Similarly, the biomass flux was constrained using the average and standard deviation of measured growth rates for batch experiments, while it was fixed to a dilution rate for chemostat experiments. The steady-state mass balance and reaction reversibility (irreversible reactions (J_{irr}) proceed in the forward direction only) constraints were also included to yield a feasible flux distribution (Eqs. S5 and S6). The resulting problem is a convex quadratic program which can be solved to global optimality.

$$\min_{w,W^{enz}} \quad \sum_{j\in J_{MFA}} \left(\frac{w_j^{exp} - w_j}{w_j^{conf}} \right)^2 + \sum_{j\in J_{GPR}} \sum_{n\in N(j)} \frac{(W_{j,n}^{enz})^2}{E_n} \qquad \text{[S1]}$$

$$s.t. \quad -\sum_{n\in N(j)} W_{j,n}^{enz} \le w_j \le \sum_{n\in N(j)} W_{j,n}^{enz} \qquad \forall j \in J_{GPR} \qquad \text{[S2]}$$

$$W_{j,n}^{enz} \ge 0 \qquad \forall j \in J_{GPR}, \forall n \in N(j) \qquad \text{[S3]}$$

$$w_j^{meas} - w_j^{std} \le w_j \le w_j^{meas} + w_j^{std} \qquad \forall j \in J_{ext} \qquad \text{[S4]}$$

$$\sum_j S_{ij} w_j = 0 \qquad \forall i \in I \qquad \text{[S5]}$$

$$w_j \ge 0 \qquad \forall j \in J_{irr} \qquad \text{[S6]}$$

The expression value for each enzyme was calculated by the sum of normalized expression levels of the genes associated to the enzyme. The reciprocal of the enzyme expression values weights the second term smaller in the objective function less the first term, and as a result the deviations in MFA estimated fluxes are more heavily penalized. We also investigated the effect of different expression value (E_n) metrics on estimated fluxes including geometric mean of log transformations, but most of the results were not significantly affected as long as they are on the same order of magnitude. However, the behavior of some isozyme knockout mutants were better predicted by the use of the sum of normalized expression levels (without log transformation) since the differences in expression levels are more apparent between highly and lowly expressed isozymes.

Predicting the metabolic flux distribution in a perturbed state using RELATCH

The reference flux distribution (w) and corresponding enzyme contribution (W^{enz}) estimated in the previous section were used to predict the flux distributions (v) in genetically or environmentally perturbed strains. Specifically, the relative changes from the reference flux distribution for reactions active in the reference state (J_{act}) was minimized in addition to the enzyme contribution (V^{enz}) for enzymes inactive in the reference state ($N \backslash N_{act}$) with a penalty (α) (Eq. S7). Here, a reaction or an enzyme was considered inactive in the reference state if its flux or contribution was less than 10^{-6} mmol gDW^{-1} hr^{-1}. The flux variables for reactions with GPR associations were constrained in the same way as above (Eqs. S8 and S9), but the contributions for active enzymes (N_{act}) was limited to the reference enzyme contributions multiplied by an increase limit (γ) (Eq. S10). A gene knockout was simulated by constraining the enzyme contributions of the associated enzymes (N_{KO}) to zero (Eq. S11), and an environmental perturbation was simulated by changing the lower bounds (v^{min} = 0 if absent, and v^{min} = $-\infty$ if present in the medium) on exchange reactions (Eq. S12). The steady-state mass balance and reaction reversibility constraints were included as well (Eqs. S13 and S14), and the biomass flux was fixed to a dilution rate for chemostat experiments. The resulting problem is also a convex quadratic program.

$$\min_{v,V^{enz}} \quad \sum_{j \in J_{act}} \left(\frac{w_j - v_j}{w_j} \right)^2 + \alpha \sum_{j \in J_{GPR}} \sum_{n \notin N_{act}(j)} V_{j,n}^{enz} \qquad \text{[S7]}$$

$$s.t. \quad -\sum_{n \in N(j)} V_{j,n}^{enz} \le v_j \le \sum_{n \in N(j)} V_{j,n}^{enz} \qquad \forall j \in J_{GPR} \qquad \text{[S8]}$$

$$V_{j,n}^{enz} \ge 0 \qquad \forall j \in J_{GPR}, \forall n \in N(j) \qquad \text{[S9]}$$

$$V_{j,n}^{enz} \le \gamma W_{j,n}^{enz} \qquad \forall j \in J_{GPR}, \forall n \in N_{act}(j) \qquad \text{[S10]}$$

$$V_{j,n}^{enz} = 0 \qquad \forall j \in J_{GPR}, \forall n \in N_{KO}(j) \qquad [S11]$$

$$v_j \geq v_j^{\min} \qquad \forall j \in J_{exch} \qquad [S12]$$

$$\sum_j S_{ij} v_j = 0 \qquad \forall i \in I \qquad [S13]$$

$$v_j \geq 0 \qquad \forall j \in J_{irr} \qquad [S14]$$

We employed two different sets of parameter values for α and γ when predicting flux distributions under different perturbations. For strains not adapted to perturbations, we used α = 10 and γ = 1.1 (tight parameter values) to restrict the activation of latent pathways and enzyme contribution increases for active enzymes. For strains well adapted to perturbations, we used α = 1 and γ = ∞ (relaxed parameter values) to allow further activation of latent pathways and enzyme contribution increases for active enzyme. To simulate cases where γ = ∞, Eq. S10 is omitted since the V^{enz} values are not constrained by the W^{enz} values.

Parameter sensitivity analysis

We performed sensitivity analysis of the two parameters (α and γ) using four *E. coli* knockout mutants (*Δpgi*, *Δppc*, *Δpta*, and *Δtpi*) before and after undergoing adaptive evolution [6]. We varied the value of α (which represents the resistance to latent pathway activation) from 10^{-3} to 10^{3} and the value of γ from (which limits enzyme contribution increases) 1 to 10 using 101 intervals on a log-scale, which resulted in a 101x101 grid, and investigated the effects of parameter values on the accuracy of flux distributions and growth rates predicted by RELATCH (Additional File 2). The results indicate that the predictions were not significantly affected by the parameter values as long as α and γ were in the same order of magnitude. For each pair of

parameter values, we calculated the sum of squared errors for predicted flux distributions in unevolved strains ($SSE_{unevolved}$, Additional File 2A) and evolved strains ($SSE_{evolved}$, Additional File 2B). In agreement with our assumption, the flux prediction errors for unevolved strains were more accurate when α is high and γ is low whereas the errors for evolved strains were more accurate when α is high and γ is low. The growth rates were also better predicted when the flux prediction errors were lower (Additional File 2C). Interestingly, the predicted growth rate gradually increased as the parameter values become less restrictive (i.e., $\alpha \rightarrow 10^{-3}$ and $\gamma \rightarrow 10$). For the *Δpgi* mutant, the predicted growth rates were sensitive to both the parameter α and γ suggesting the major bottleneck that needs to be overcome during evolution includes activation of latent pathways as well as increases in flux through already active enzymes. For the *Δppc* and *Δtpi* mutants, the predicted growth rates were more sensitive to the parameter α suggesting that the activation of latent pathways is needed to improve growth rates. The result for *Δpta* mutants is not shown in Additional File 2 since the flux differences between unevolved and evolved were not significant both experimentally and computationally.

Strains and growth phenotyping

The Keio collection of in-frame single-gene deletion strains [8] and *E. coli* K-12 BW25113 (the parent strain of the Keio collection) were used to generate the growth rate dataset shown in Fig. 3 and Additional File 7. The kanamycin resistant gene (kan) was removed from 22 single knockout mutants (*aceA*, *aceB*, *aceE*, *ackA*, *fumC*, *gnd*, *ldhA*, *maeA*, *maeB*, *mdh*, *ndh*, *nuoF*, *pck*, *ppc*, *sdhA*, *sthA*, *sucA*, *sucC*, *talA*, *tktA*, *tktB*, and *zwf*) before screening mutant phenotypes. Growth phenotyping experiments were conducted for these 22 mutant and parental strains on 2 g/liter glucose-supplemented M9 minimum medium (6.8 g of Na_2HPO_4, 3 g of KH_2PO_4, 0.5 g of NaCl, 1 g of NH_4Cl, 2 ml of 1M $MgSO_4$, and 100 μl of 1 M $CaCl_2$ per liter) in triplicate at 37°C using a

Tecan Infinite 200 microplate reader (Tecan Group Ltd., Switzerland). Strains were pre-cultured overnight in LB medium, and washed before being resuspended in fresh glucose M9 medium with the starting optical density at 600 nm (OD_{600}) around 0.05. The OD_{600} value was measured by the microplate reader every 15 minutes for 36 hours, while the plates were continuously incubated and shaken. The maximum growth rate was calculated by log-linear regression of OD_{600} versus time, and the average and standard deviation of triplicates were calculated.

References

1. Feist AM, Henry CS, Reed JL, Krummenacker M, Joyce AR, Karp PD, Broadbelt LJ, Hatzimanikatis V, Palsson BO: **A genome-scale metabolic reconstruction for Escherichia coli K-12 MG1655 that accounts for 1260 ORFs and thermodynamic information.** *Mol Syst Biol* 2007, **3:**121.
2. Mo ML, Palsson BO, Herrgard MJ: **Connecting extracellular metabolomic measurements to intracellular flux states in yeast.** *BMC Syst Biol* 2009, **3:**37.
3. Oh YK, Palsson BO, Park SM, Schilling CH, Mahadevan R: **Genome-scale reconstruction of metabolic network in Bacillus subtilis based on high-throughput phenotyping and gene essentiality data.** *J Biol Chem* 2007, **282:**28791-28799.
4. Lewis NE, Hixson KK, Conrad TM, Lerman JA, Charusanti P, Polpitiya AD, Adkins JN, Schramm G, Purvine SO, Lopez-Ferrer D, et al: **Omic data from evolved E. coli are consistent with computed optimal growth from genome-scale models.** *Mol Syst Biol* 2010, **6:**390.
5. Shlomi T, Berkman O, Ruppin E: **Regulatory on/off minimization of metabolic flux changes after genetic perturbations.** *Proc Natl Acad Sci U S A* 2005, **102:**7695-7700.
6. Fong SS, Nanchen A, Palsson BO, Sauer U: **Latent pathway activation and increased pathway capacity enable Escherichia coli adaptation to loss of key metabolic enzymes.** *J Biol Chem* 2006, **281:**8024-8033.
7. Bologna FP, Campos-Bermudez VA, Saavedra DD, Andreo CS, Drincovich MF: **Characterization of Escherichia coli EutD: a phosphotransacetylase of the ethanolamine operon.** *J Microbiol* 2010, **48:**629-636.
8. Baba T, Ara T, Hasegawa M, Takai Y, Okumura Y, Baba M, Datsenko KA, Tomita M, Wanner BL, Mori H: **Construction of Escherichia coli K-12 in-frame, single-gene knockout mutants: the Keio collection.** *Mol Syst Biol* 2006, **2:**2006 0008.
