# Supplementary material for: RELATCH: relative optimality in metabolic networks explains robust metabolic and regulatory responses to perturbations
Source: Genome Biol. 2012 Sep 26;13(9):R78. doi: 10.1186/gb-2012-13-9-r78 (PMC3506949; doi:10.1186/gb-2012-13-9-r78)
Supplement: Additional File 2 — Supplementary Figure S1. Sensitivity analysis of parameters using E. coli knockout strains (Δpgi, Δppc, and Δtpi) before and after adaptive evolution. [file gb-2012-13-9-r78-S2.PDF]

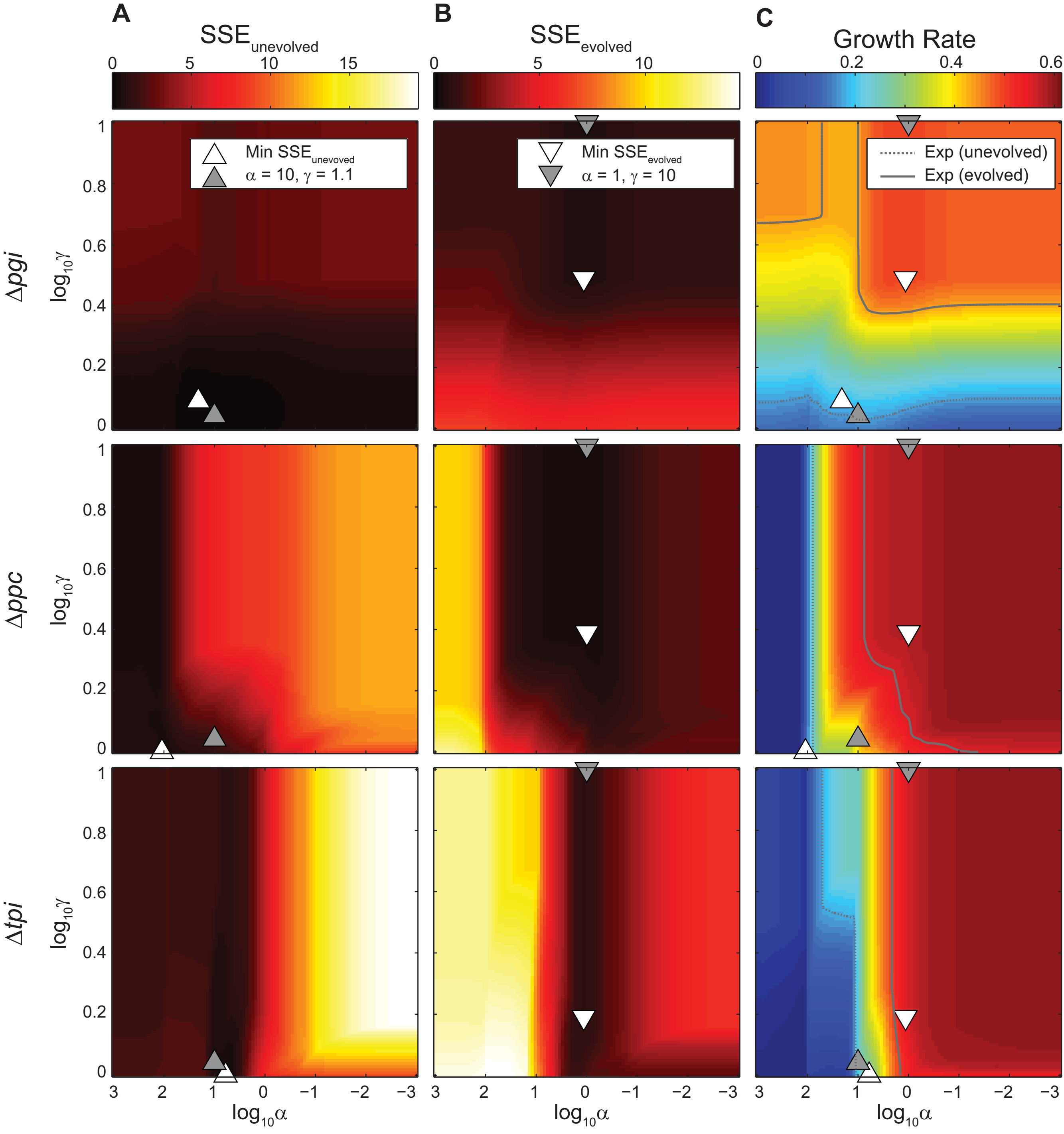

Supplementary Figure S1. Sensitivity analysis of parameters (α and γ) using *E. coli* knockout strains (*Δpgi*, *Δppc*, and *Δtpi*) before and after adaptive evolution. The colors correspond to (A) the sum of squared errors per flux for metabolic flux predictions in unevolved mutant strains ($SSE_{unevolved}$), (B) the sum of squared errors per flux for metabolic flux predictions in evolved mutant strains ($SSE_{evolved}$), and (C) the growth rate predictions by RELATCH using different parameter values of α (x-axis, log-scale) and γ (y-axis, log-scale). For each set of parameter values of α and γ, the resulting RELATCH solution was compared to the MFA flux values and the SSE was calculated using Eq 6. The set of parameters with the minimum SSE (compared to all other parameter values) is shown as white triangles (upward triangle = unevolved strain; downward triangle = evolved strain). Grey triangles show the parameters used in the Results section (upward triangle = unevolved strain; downward triangle = evolved strain). The SSE values at the relaxed parameters (α = 1 and γ = ∞) were the same as the SSE values at α = 1 and γ = 10. Units for growth rate are hr^{-1} and units for SSE are ($mmol^2\ gDW^{-2}\ hr^{-2}$).
