# Supplementary material for: RELATCH: relative optimality in metabolic networks explains robust metabolic and regulatory responses to perturbations
Source: Genome Biol. 2012 Sep 26;13(9):R78. doi: 10.1186/gb-2012-13-9-r78 (PMC3506949; doi:10.1186/gb-2012-13-9-r78)
Supplement: Additional File 3 — Supplementary Table S1. Comparison of predicted and experimentally measured values of growth, substrate uptake, and product secretion rates for four E. coli mutants before adaptive evolution. [file gb-2012-13-9-r78-S3.PDF]

Table S1. Comparison of predicted and experimentally measured values of growth, substrate uptake, and product secretion rates for four *E. coli* mutants before adaptive evolution.

Strain	Method	Growth Rate (hr^{-1})	Glucose Uptake (mmol/gDW/hr)	Acetate Production (mmol/gDW/hr)	Pyruvate Production (mmol/gDW/hr)
Wildtype	Experiment	0.63	8.80	4.50	0.00
Δpgi	Experiment	0.17	2.30	0.10	0.00
	FBA	0.84	8.80	0.00	0.00
	MOMA	0.46	8.63	4.57	0.00
	ROOM	0.60	8.47	4.44	0.00
	RELATCH	0.18	2.54	0.00	0.00
Δppc	Experiment	0.22	3.00	1.10	0.00
	FBA	0.84	8.80	0.00	0.00
	MOMA	0.43	9.09	4.24	0.02
	ROOM	0.60	8.52	4.44	0.00
	RELATCH	0.40	5.66	2.11	0.00
Δpta	Experiment	0.58	9.10	0.60	4.30
	FBA	0.85	8.80	0.00	0.00
	MOMA	0.52	8.83	3.68	0.13
	ROOM	0.60	8.47	4.44	0.00
	RELATCH	0.61	9.10	0.00	4.99
Δtpi	Experiment	0.18	2.70	0.20	0.00
	FBA	0.82	8.80	0.00	0.00
	MOMA	0.34	13.20	5.08	0.58
	ROOM	0.60	15.54	4.61	0.00
	RELATCH	0.21	2.92	0.00	0.00
