# Supplementary material for: RELATCH: relative optimality in metabolic networks explains robust metabolic and regulatory responses to perturbations
Source: Genome Biol. 2012 Sep 26;13(9):R78. doi: 10.1186/gb-2012-13-9-r78 (PMC3506949; doi:10.1186/gb-2012-13-9-r78)
Supplement: Additional File 4 — Supplementary Table S2. Comparison of predicted and experimentally measured values of growth, substrate uptake, and product secretion rates for four E. coli mutants after adaptive evolution. [file gb-2012-13-9-r78-S4.PDF]

Table S2. Comparison of predicted and experimentally measured values of growth, substrate uptake, and product secretion rates for four *E. coli* mutants after adaptive evolution. The experimental values for two parallel evolved mutants are shown and indicated as E1 and E2.

Strain	Method	Growth Rate (hr^{-1})	Glucose Uptake (mmol/gDW/hr)	Acetate Production (mmol/gDW/hr)	Pyruvate Production (mmol/gDW/hr)
Wildtype	Experiment	0.63	8.80	4.50	0.00
Δpgi	Experiment (E1)	0.34	5.80	2.60	0.00
	Experiment (E2)	0.53	5.60	0.00	0.00
	FBA	0.84	8.80	0.00	0.00
	MOMA	0.46	8.63	4.57	0.00
	ROOM	0.60	8.47	4.44	0.00
	RELATCH	0.50	5.64	0.00	0.00
Δppc	Experiment (E1)	0.55	8.10	2.20	0.00
	Experiment (E2)	0.56	7.80	2.20	0.00
	FBA	0.84	8.80	0.00	0.00
	MOMA	0.43	9.09	4.24	0.02
	ROOM	0.60	8.52	4.44	0.00
	RELATCH	0.58	7.92	3.19	0.00
Δpta	Experiment (E1)	0.64	10.30	0.70	4.60
	Experiment (E2)	0.66	8.60	0.70	2.80
	FBA	0.85	8.80	0.00	0.00
	MOMA	0.52	8.83	3.68	0.13
	ROOM	0.60	8.47	4.44	0.00
	RELATCH	0.61	9.29	0.00	5.21
Δtpi	Experiment (E1)	0.51	7.80	1.00	0.00
	Experiment (E2)	0.49	7.30	0.90	0.00
	FBA	0.82	8.80	0.00	0.00
	MOMA	0.34	13.20	5.08	0.58
	ROOM	0.60	15.54	4.61	0.00
	RELATCH	0.56	7.77	3.19	0.00
