# Supplementary material for: RELATCH: relative optimality in metabolic networks explains robust metabolic and regulatory responses to perturbations
Source: Genome Biol. 2012 Sep 26;13(9):R78. doi: 10.1186/gb-2012-13-9-r78 (PMC3506949; doi:10.1186/gb-2012-13-9-r78)
Supplement: Additional File 5 — Supplementary Figure S2. Sensitivity analysis of data used to generate reference flux distributions. [file gb-2012-13-9-r78-S5.PDF]

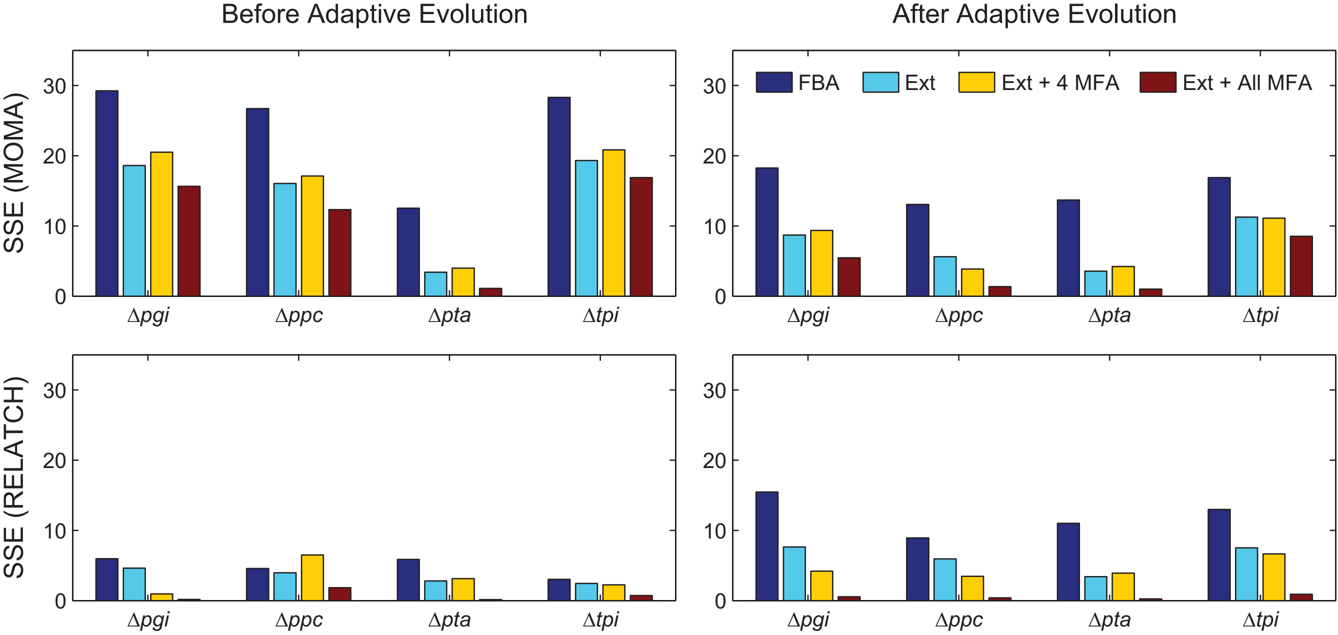

Supplementary Figure S2. Sensitivity analysis of data available to estimate the reference flux distributions. Four different scenarios with different availability of experimental data for the reference state were considered to investigate the robustness of predictions for the perturbed state. (1) A pFBA solution was used as the reference state (where growth rate is first maximized and then the flux usage is minimized); (2) External flux measurements (glucose uptake, acetate secretion, pyruvate secretion, and growth rates) were used; (3) External flux measurements and a subset of MFA estimates (phosphoglucose isomerase for glycolysis, glucose-6-phosphate dehydrogenase for the pentose-phosphate pathway, citrate synthase for the TCA cycle, and pyruvate dehydrogenase) were used, or (4) External flux measurements and all MFA estimates were used to calculate the reference flux distribution. For scenarios (2) - (4), the reference flux distribution was estimated using Eqs. S1 - S6 with the same gene expression data and the specified set of measured fluxes. The subset of MFA estimates included in (3) was chosen based on a MFA study for *B. subtilis* (Fischer and Sauer, 2005) to represent cases with limited MFA data. For each scenario, the sum of squared errors per flux (SSE) was calculated for MOMA and RELATCH predictions for four *E. coli* mutants before and after adaptive evolution (ROOM predictions were very similar to MOMA predictions, data not shown).
