# Supplementary material for: RELATCH: relative optimality in metabolic networks explains robust metabolic and regulatory responses to perturbations
Source: Genome Biol. 2012 Sep 26;13(9):R78. doi: 10.1186/gb-2012-13-9-r78 (PMC3506949; doi:10.1186/gb-2012-13-9-r78)
Supplement: Additional File 6 — Supplementary Figure S3. Sensitivity analysis of metabolic network models. [file gb-2012-13-9-r78-S6.PDF]

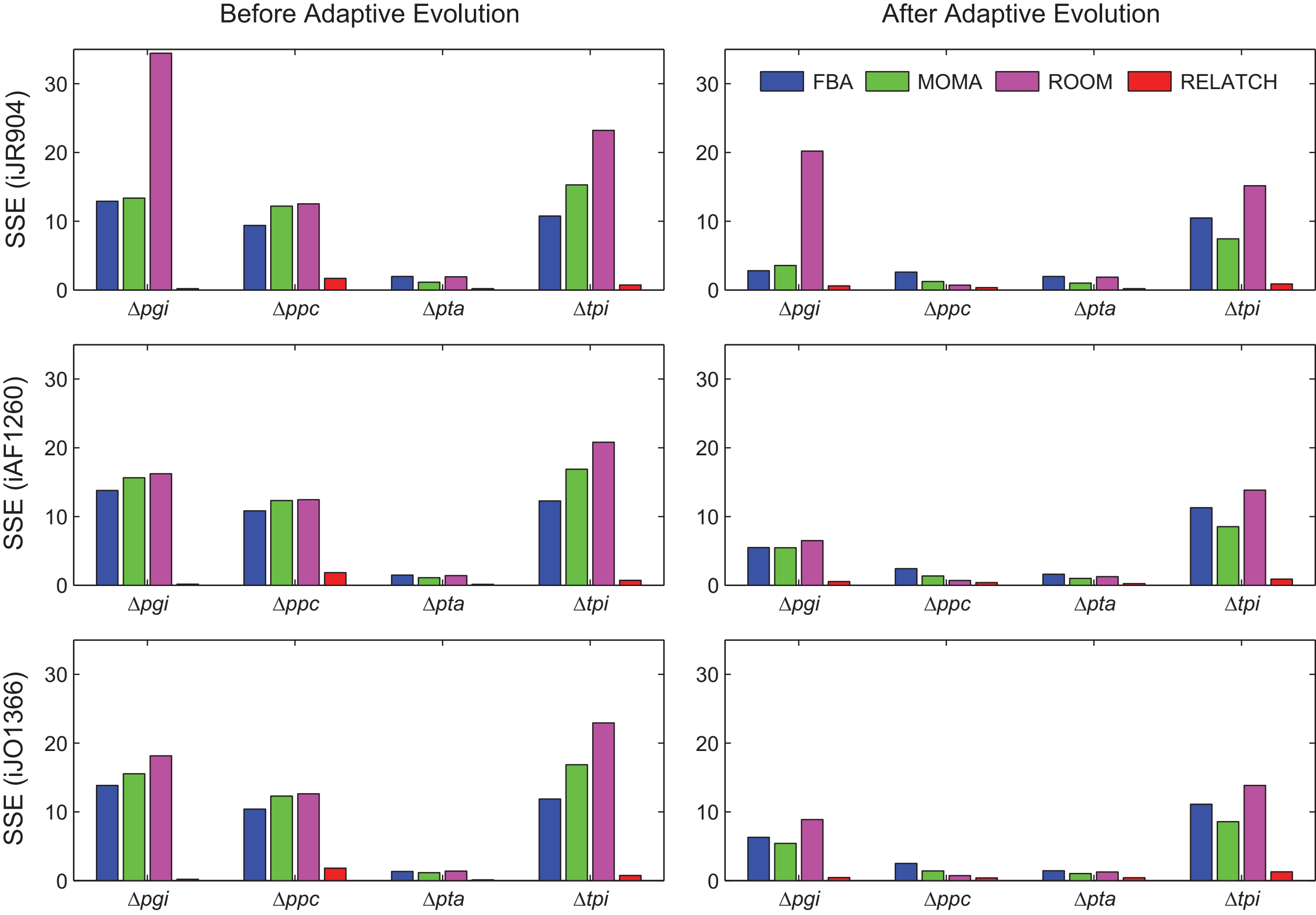

Supplementary Figure S3. Sensitivity analysis of metabolic network models. Three different *E. coli* metabolic models (iJR904, iAF1260, and iJO1366) were used to investigate the robustness of predictions to a metabolic model. For each model, the sum of squared errors per flux (SSE) was calculated for FBA, MOMA, ROOM, and RELATCH predictions for four *E. coli* mutants before and after adaptive evolution.
