# Supplementary material for: RELATCH: relative optimality in metabolic networks explains robust metabolic and regulatory responses to perturbations
Source: Genome Biol. 2012 Sep 26;13(9):R78. doi: 10.1186/gb-2012-13-9-r78 (PMC3506949; doi:10.1186/gb-2012-13-9-r78)
Supplement: Additional File 7 — Supplementary Table S3. Growth rate predictions for 22 single knockout E. coli mutants. [file gb-2012-13-9-r78-S7.PDF]

Table S3. Comparison of experimentally measured and predicted growth rates (hr^{-1}) for 22 single knockout *E. coli* mutants.

Mutant	Exp.	FBA	MOMA	ROOM	RELATCH
aceA	0.55	0.80	0.60	0.58	0.60
aceB	0.63	0.80	0.60	0.58	0.60
aceE	0.43	0.78	0.21	0.61	0.52
ackA	0.56	0.80	0.60	0.58	0.59
fumC	0.67	0.80	0.60	0.58	0.60
gnd	0.61	0.79	0.54	0.58	0.42
ldhA	0.57	0.80	0.60	0.58	0.60
maeA	0.49	0.80	0.60	0.59	0.60
maeB	0.61	0.80	0.60	0.58	0.60
mdh	0.57	0.78	0.59	0.60	0.59
ndh	0.54	0.80	0.53	0.58	0.45
nuoF	0.57	0.70	0.36	0.58	0.32
pck	0.47	0.80	0.60	0.59	0.60
ppc	0.18	0.79	0.43	0.60	0.00
sdhA	0.73	0.77	0.51	0.58	0.59
sthA	0.59	0.80	0.60	0.58	0.60
sucA	0.45	0.79	0.55	0.58	0.55
sucC	0.61	0.79	0.56	0.58	0.57
talA	0.55	0.80	0.60	0.58	0.58
tktA	0.50	0.80	0.60	0.58	0.54
tktB	0.66	0.80	0.60	0.58	0.57
zwf	0.48	0.79	0.51	0.59	0.38
