# Supplementary material for: RELATCH: relative optimality in metabolic networks explains robust metabolic and regulatory responses to perturbations
Source: Genome Biol. 2012 Sep 26;13(9):R78. doi: 10.1186/gb-2012-13-9-r78 (PMC3506949; doi:10.1186/gb-2012-13-9-r78)
Supplement: Additional File 10 — Supplementary Figure S4. Comparison of metabolic flux predictions using RELATCH and MOMA for knockout mutants of S. cerevisiae and B. subtilis. [file gb-2012-13-9-r78-S10.PDF]

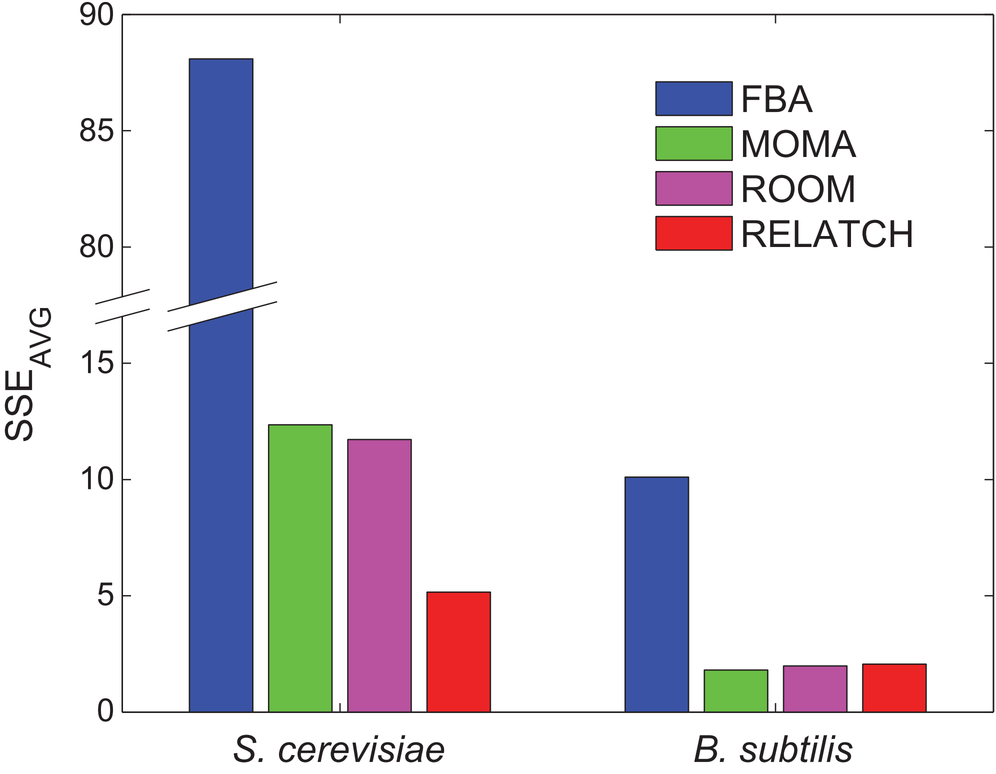

Supplementary Figure S4. Comparison of metabolic flux predictions for 25 of 35 *S. cerevisiae* mutants and 27 of 63 *B. subtilis* mutants (only mutants involving genes without isozymes are compared). The average of the sum of squared errors per flux (SSE_{AVG}) across the mutants is shown for FBA, MOMA, ROOM, and RELATCH.
