# Supplementary material for: RELATCH: relative optimality in metabolic networks explains robust metabolic and regulatory responses to perturbations
Source: Genome Biol. 2012 Sep 26;13(9):R78. doi: 10.1186/gb-2012-13-9-r78 (PMC3506949; doi:10.1186/gb-2012-13-9-r78)
Supplement: Additional File 11 — Supplementary Figure S5. Comparison of MFA estimated fluxes and predictions by RELATCH for E. coli strains grown on galactose. [file gb-2012-13-9-r78-S11.PDF]

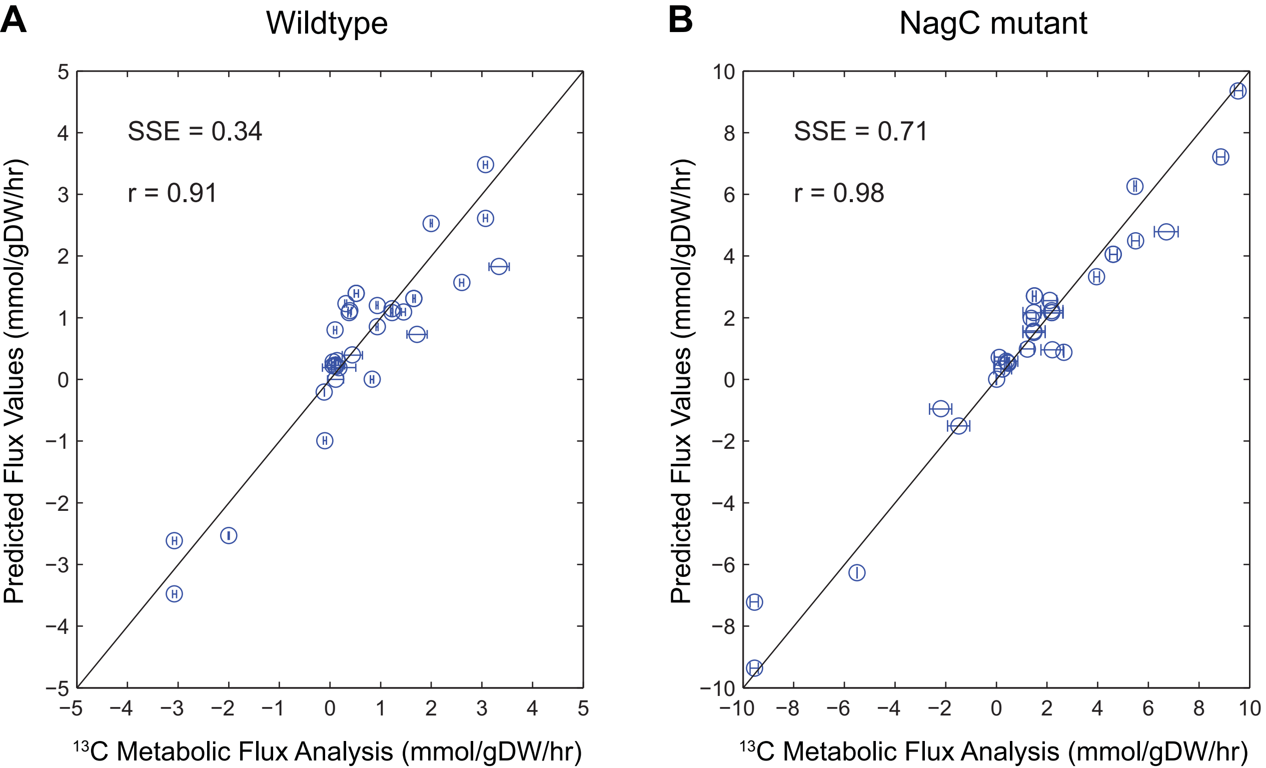

Supplementary Figure S5. Comparison of ^{13}C MFA estimated fluxes and predictions by RELATCH for (A) wildtype *E. coli* strain and (B) NagC transcription factor knockout *E. coli* strain grown on galactose. The sum of squared errors per flux (SSE) and the Pearson's correlation coefficient (r) are shown for each strain, and the error bars indicate the confidence intervals from ^{13}C MFA.
